# Dexamethasone reduces emesis after major gastrointestinal surgery (DREAMS)

**DOI:** 10.1186/1745-6215-14-249

**Published:** 2013-08-12

**Authors:** Emma Hamilton, Reena Ravikumar, David Bartlett, Elizabeth Hepburn, Mei-Ju Hwang, Nazzia Mirza, Sandeep S Bahia, Anthony Wilkey, Helen Bodenham Chilton, Kelly Handley, Laura Magill, Dion Morton

**Affiliations:** 1 West Midlands Deanery/West Midlands Research Collaborative, Birmingham, UK; 2 London Deanery / West Midlands Research Collaborative, Birmingham, UK; 3 Biomedical Research Unit and Centre for Liver Research, University of Birmingham, Birmingham, UK; 4 Centre for Liver Research, Infection and Immunity University of Birmingham, Birmingham, UK; 5 Department of Surgery, New Cross Hospital, Wolverhampton, UK; 6 St Georges Hospital, London, UK; 7 Department of Anaesthetics, University Hospitals Birmingham, Birmingham, UK; 8 Birmingham Clinical Trials Unit, University of Birmingham, Birmingham, UK; 9 Academic Department of Surgery, University Hospitals Birmingham, Birmingham, UK

**Keywords:** Anti-emetics, Colorectal surgery, Dexamethasone, Enhanced recovery, Gastrointestinal/surgery, Post-operative nausea and vomiting

## Abstract

**Background:**

Postoperative nausea and vomiting is one of the most common complications affecting patients after surgery and causes significant morbidity and increased length of hospital stay. It is accepted that patients undergoing surgery on the bowel are at a higher risk. In the current era of minimally invasive colorectal surgery combined with enhanced recovery, reducing the incidence and severity of postoperative nausea and vomiting is particularly important. Dexamethasone is widely, but not universally used. It is known to improve appetite and gastric emptying, thus reduce vomiting. However, this benefit is not established in patients undergoing bowel surgery, and dexamethasone has possible side effects such as increased risk of wound infection and anastomotic leak that could adversely affect recovery.

**Design:**

DREAMS is a phase III, double-blind, multicenter, randomized controlled trial with the primary objective of determining if preoperative dexamethasone reduces postoperative nausea and vomiting in patients undergoing elective gastrointestinal resections. DREAMS aims to randomize 1,350 patients over 2.5 years.

Patients undergoing laparoscopic or open colorectal resections for malignant or benign pathology are randomized between 8 mg intravenous dexamethasone and control (no dexamethasone). All patients are given one additional antiemetic at the time of induction, prior to randomization. Both the patient and their surgeon are blinded as to the treatment arm.

Secondary objectives of the DREAMS trial are to determine whether there are other measurable benefits during recovery from surgery with the use of dexamethasone, including quicker return to oral diet and reduced length of stay. Health-related quality of life, fatigue and risks of infections will be investigated.

**Trial registration:**

ISRCTN21973627

## Background

Postoperative nausea and vomiting (PONV) is one of the most common complications affecting patients after surgery. The pathophysiology of PONV is multifactorial, but as patients undergoing colorectal surgery are exposed to various causative agents in addition to the physical factors of bowel manipulation predisposing to ileus, they are at a substantially higher risk of this problem. Following surgical intervention, patients view PONV as a very undesirable outcome, even more unpleasant than pain [1]. It causes significant morbidity, delays nutrition and increases the length of stay in hospital. Given that over 60,000 colorectal operations are performed annually in the UK alone, PONV has significant medical and cost implications to healthcare.

### Pathophysiology of PONV

The pathophysiology of nausea and vomiting is complex. The vomiting centre in the medulla receives stimulation from various sources, including the chemoreceptor trigger zone, enteric vagal nerve afferents, the vestibular system and the cerebral cortex. The multifactorial mechanisms underlying PONV include stimulation of dopamine and 5-hydroxytryptamine type 3 receptors in the chemoreceptor trigger zone by noxious substances such as opiates and anaesthetic agents, and the stimulation of gut chemoreceptors and stretch receptors. Despite advances in antiemetic agents, anaesthetic agents and minimally invasive surgical techniques, PONV remains a significant and persistent problem. Other risk factors that increase the likelihood of PONV are female gender, non-smoking status, history of motion sickness, childhood and young adulthood, and increasing length of surgery [2]. Intra-abdominal and laparoscopic surgery has been implicated as having an increased risk of PONV. A comparison of predictive models for PONV [3] found that intra-abdominal surgery had an odds ratio of 1.23 (n = 217) but this included patients who underwent gynecological laparoscopy. Laparoscopic cholecystectomy had an odds ratio of 2.85 (n = 42).

### Clinical relevance of PONV

After surgery, the overall incidence of PONV is 30%, and it is up to 70% in high-risk patients [2]. In a study investigating the 10 most undesirable postoperative outcomes, vomiting ranked first, gagging on the endotracheal tube second, and postoperative pain third [1]. Nausea was ranked fourth. Although mortality rates are rarely affected, PONV can cause significant morbidity including dehydration, electrolyte disturbance, delayed return to diet and aspiration pneumonia. Delayed recovery in the hospital setting predisposes to serious and life-threatening complications such as hospital-acquired pneumonia and thromboembolic events (deep venous thrombosis and pulmonary embolism). The delay in patients resuming an oral diet affects nutrition and subsequent general well-being, predisposing to tissue breakdown, pressure sores, wound infection, fatigue, weakness and delay in mobilization.

In the current era of minimally invasive colorectal surgery combined with enhanced recovery protocols to optimize recovery and reduce the length of patient hospital stay, reducing the incidence and severity of PONV is particularly important. The cost implications related to prolonged hospital stay and the additional costs of further complications affect both open and laparoscopic surgery.

### Dexamethasone for PONV

Dexamethasone is extensively used in all types of surgery to reduce PONV (Table 1). In practice, dexamethasone is less widely used for control of PONV by anesthetists in gastrointestinal patients, perhaps because of a lack of proven efficacy in these patients. However, its use is advocated in some enhanced recovery programs (ERAS) to improve recovery after colorectal surgery [4-6].

**Table 1:** Literature review of use of dexamethasone for PONV

**Author**	**Year**	**n**	**Type of surgery**	**Findings**	**Reference**
Apfel *et al.*	2004	5,199	Gynecological, Trauma, Abdominal, Otolaryngology	Ondansetron, dexamethasone, and droperidol each reduced the risk of PONV by 26%	[7]
Wallenborn *et al.*	2006	3,140	Gynecological, Trauma Abdominal, Otolaryngology	Metoclopramide plus dexamethasone is an effective, safe and cheap way to prevent PONV	[8]
Zagar-Shostari *et al.*	2009	60	Colorectal	Dexamethasone in an enhanced recovery protocol gives a significant reduction in early postoperative fatigue, and an attenuated peritoneal cytokine response	[9]
Kirdak *et al.*	2008	30	Colorectal	Dexamethasone has no significant effect on reducing postoperative pain, inflammatory response or PONV	[10]
Weren and Demeere	2008	118	Abdominal and Gynecological	Steroids are mostly effective in the prevention of late PONV (rather than early).	[11]
Hans *et al.*	2006	32	Abdominal	After dexamethasone, blood glucose levels increase in both patients without diabetes and those with type 2 diabetes undergoing abdominal surgery. In patients without diabetes blood sugar levels rose to a maximum of 10 mmol / litre	[12]
Coloma *et al.*	2001	80	Anorectal	Reduction in time to ‘home readiness’ in ambulatory surgery	[13]
Gautam *et al.*	2008	150	Laparoscopic cholecystectomy	Combination of ondansetron and dexamethasone is better than each drug alone	[14]
Tiippana *et al.*	2008	160	Laparoscopic cholecystectomy	Dexamethasone decreased the need for opiates	[15]
Bianchin *et al.*	2007	80	Laparoscopic cholecystectomy	Reduced PONV. No change in pain or time to discharge.	[16]
Wang *et al.*	1999	90	Laparoscopic cholecystectomy	Dexamethasone significantly decreased the incidence of PONV	[17]
Sanchez- Rodriguez *et al.*	2010	210	Laparoscopic cholecystectomy	Dexamethasone significantly reduced PONV at 0, 6, 12 hours and reduced postoperative pain and fatigue	[18]
Mathiesen *et al.*	2009	116	Gynecological	Reduced PONV. Combinations of paracetamol, pregabalin and dexamethasone did not reduce morphine consumption.	[19]
Biswas *et al.*	2003	160	Gynecological	Ondansetron plus dexamethasone is most effective in preventing PONV. Results comparable for single agents.	[20]
Yursek *et al.*	2003	60	Gynecological	Ondansetron, but not dexamethasone, prevented PONV by 3 h post operation	[21]
Wang *et al.*	2000	90	Gynecological	Dexamethasone significantly decreases the incidence of PONV	[22]
McKean *et al.*	2006	72	Otolaryngology	Significant decrease in PONV and pain scores	[23]
Mathew *et al.*	2004	210	Pediatrics	Dexamethasone is effective for the prevention of PONV after strabismus repair in children	[24]

PONV:postoperative nausea and vomiting.

Its precise mechanism of action is unknown but it has been proposed that the antiemetic properties arise due to activation of glucocorticoid receptors in the medulla [25], or by inhibiting central production of prostaglandins or inhibiting the release of endogenous opioids [26]. It is also known to improve appetite [27], and in combination with reduced nausea and vomiting, aids early recovery. Glucocorticoids can also reduce pain by suppression of bradykinin and neuropeptides from nerve endings [26].

Dexamethasone does have potential theoretical side effects such as increased risks of wound infections and anastomotic leaks that could adversely affect recovery, but a systematic review of 51 studies using a single dose of methylprednisolone in cardiac, general and trauma surgical patients found no significant increase in adverse events [28].

### Dexamethasone use in abdominal surgery

In 2008, a systematic review and meta-analysis of 17 randomized clinical trials looking at preoperative dexamethasone in patients undergoing laparoscopic cholecystectomy was published [29]. This review suggested that, regardless of co-intervention (the administration of other antiemetics in the control arm), dexamethasone reduced the incidence of nausea and vomiting. Small studies investigating use of dexamethasone to reduce PONV in many other fields of surgery have been undertaken (Table 1). However, there are only two single center colorectal trials, totaling 100 patients, that have assessed this guidance in patients undergoing gastrointestinal surgery [9,10]. Interestingly, one of these trials [9] measured cytokine levels in peritoneal drain fluid following colorectal surgery and found significantly reduced levels of IL-6, a pro-inflammatory cytokine produced by T-cells and macrophages. There was a significant reduction in IL-13 (a cytokine involved in type 2 T-helper cell responses), which is a stimulator of inflammation and tissue remodeling at sites of T-helper type 2 inflammation. There was also a reduction in plasma levels of IL-6 and IL-8, another major stimulator of the inflammatory response. These changes in cytokine levels correlated with improved fatigue scores.

The well-conducted Apfel *et al*. study from 2004 randomized over 4,000 patients and assessed 64 different combinations of anesthetic measures [7]. They concluded that dexamethasone was effective in preventing PONV. However, only 11% of patients in this study underwent general surgical procedures and only a small fraction of these underwent major colorectal surgery. Patients undergoing colorectal surgery comprise an especially high-risk population for PONV, and may gain considerable benefit from dexamethasone. As yet, there is little evidence to support its use in colorectal surgery and a prospective multicenter trial is required.

Apfel *et al*. also demonstrated that the type of volatile anesthetic used had no effect on the incidence of nausea and vomiting. The additional antiemetic given at induction will not be standardized in the proposed trial. This will maximize the generalizability of the findings.

### The need for DREAMS – a large, multicenter, randomized trial

Randomized controlled trials have shown a reduction in PONV among patients undergoing various other types of surgery who are given dexamethasone. In the absence of good evidence, dexamethasone is variably used in patients undergoing colorectal surgery. We have surveyed six major colorectal units in the West Midlands region and found that 25% of colorectal patients currently receive dexamethasone. The Dexamethasone Reduces Emesis After Major Gastrointestinal Surgery (DREAMS) trial seeks to determine the effectiveness of dexamethasone for colorectal patients. No adequately powered multicenter trial has been undertaken to conclusively assess the effect of dexamethasone on PONV in patients undergoing bowel surgery. The aim of DREAMS is to evaluate the potential benefits of a single dose of dexamethasone at induction for patients undergoing colorectal surgery. The findings from DREAMS may also give an indication of its appropriate use inside and outside of ERAS, although this would require further confirmatory research.

## Methods

### Trial design

The DREAMS trial is a two stage trial, comprising a pilot study followed by a large, phase III double-blind, multicenter, randomized controlled trial comparing the effects of a single dose of 8 mg intravenous dexamethasone on patient recovery after major gastrointestinal surgery. Centers in the UK undertaking major elective gastrointestinal surgery will be eligible to take part.

### Ethical approval

Full ethical approval has been gained from the East Midlands Research Ethics Committee (ref: 10/H0402/77).

### Randomized comparison

Patients undergoing laparoscopic or open gastrointestinal resections will be randomized, in a 1:1 ratio, between 8 mg intravenous dexamethasone and control. All patients must be given one additional antiemetic prior to randomization; however, this must not be dexamethasone.

### Inclusion criteria

All patients undergoing elective open and laparoscopic small and large bowel operations for malignant or benign pathology will be eligible for inclusion. This includes small and large bowel resections, defunctioning stomas and closures of stomas.

### Exclusion criteria

The exclusion criteria are:

•diabetes or hyperglycemia

•active gastric ulceration confirmed endoscopically

•acute angle glaucoma

•bowel obstruction

•pregnancy

•under 18 years of age

•patients currently taking any form of steroid medication, or have taken steroids in the last 3 months (except steroidal inhalers, suppositories, pessaries, eye-drops, one-off local injections to a joint and topical preparations)

•a known adverse reaction to dexamethasone

•patients taking immunosuppressive agents including, but not limited to, methotrexate and antiretrovirals.

On admission, patients will have blood glucose levels checked to exclude undiagnosed hyperglycemia. Patients unable or unwilling to give written informed consent for the study are also excluded.

### Primary outcome measure

The primary objective of the main phase of DREAMS will be to determine whether preoperative dexamethasone reduces PONV within the first 24 hours in patients undergoing elective gastrointestinal resections. The primary outcome measure will be the number of episodes of vomiting within 24 hours post-surgery, recorded prospectively on trial care charts.

### Secondary outcome measure

Comparisons will be made to assess the effect of dexamethasone on the following outcomes:

•nausea and vomiting measured objectively by the frequency of use of ‘as required’ postoperative antiemetics

•nausea measured subjectively by the validated PONV Intensity Scale

•fatigue measured using a validated assessment score (Functional Assessment of Chronic Illness Therapy-Fatigue (FACIT-F) questionnaire)

•time to tolerating oral diet

•length of hospital stay

•health-related quality of life (as measured by the EuroQol EQ-5D)

•incremental cost-effectiveness of dexamethasone compared to standard care

•infection rates and healing complications within 30 days of surgery.

Infections and complications specifically assessed include wound infections, dehiscence, anastomotic leaks, intra-abdominal collections, urinary or chest infections, and new onset diabetes.

### Randomization

Secure internet-based central randomization is available 24 hours a day and will ensure concealment of treatment allocation. Each center and each randomizer will be provided with a unique log-in and password to enable them to access the online randomization service. Participants will be randomized into the DREAMS trial, in a 1:1 ratio, to 8 mg intravenous dexamethasone or to control (no dexamethasone). A ‘minimization’ procedure using a computer-based algorithm will be used to avoid chance imbalances in important stratification variables. The stratification variables are gender, smoking status, American Society of Anesthesiologists (ASA) score, open and laparoscopic surgical cases, intended postoperative analgesia (intravenous patient-controlled analgesia (PCA), epidural infusion or epidural PCA), and patients within and outside of an ERAS pathway. For trial purposes, ERAS is defined as any recovery process that is expedient over traditional patient care to include but not exclusive to earlier feeding and mobilization. The definition of ERAS is specifically left broad to recognize the differences in implementation of this system across the various units.

### Power calculation

The DREAMS trial aims to recruit a total of 1,350 patients from around 30 colorectal units. With 1,350 patients randomized, DREAMS would have 90% power to detect a 24% proportional reduction (37% to 28%) in episodes of PONV in patients taking dexamethasone after undergoing gastrointestinal surgery. This number includes an additional 10% for crossover and dropout. Subgroup analyses will be undertaken for variables for which the randomization is stratified (for example, enhanced versus non-enhanced recovery pathway, PCA versus epidural) using standard tests for interactions. Additional exploratory analyses will also be undertaken to investigate, for example, any correlation between operative length and risk of nausea and vomiting. Analysis of the study will be on an intention-to-treat basis.

### Feasibility study

A lead-in phase II feasibility study was undertaken before the full phase III trial; it was planned that these patients would be included in the phase III trial analysis. The aim of the pilot study was to inform the processes to be used in the main trial. Three main areas were addressed: the strategies used for the identification of eligible patients; defining the patient pathway; and evaluation of the case report forms. In addition to this, the process of blinding in theater required development.

Two forms of treatment allocation were evaluated in the feasibility study, by telephone and by email. Randomization was done online (detailed below). But it was found that email treatment allocation was difficult to quality control because of the large number of anesthetists supporting the trial. After the first 100 patients, telephone randomization was used, as this allowed the trial team to check the patient was anesthetized before the allocation was released, thus reducing a potential source of bias.

The patient pathway and ability of centers to recruit was assessed because DREAMS requires support from both surgeons and anesthetists to maximize recruitment and to ensure that all eligible patients are approached. Recruitment was assessed on a monthly basis. The initial aim was that by the end of the pilot study (measured as six months after the study opened to recruitment), one patient per center per week should be recruited from five open centers. Within DREAMS, potential patients can be identified from a number of clinical settings; in the pilot, the percentage of eligible patients screened for entry into the trial was recorded and the subsequent acceptance rate was a measure of the success of the process for patient identification and randomization.

Across all sites there will be variations in the patient pathway as the logistics and timings of patients being seen in the outpatient clinic, pre-admissions and admissions will differ from trust to trust. As part of the feasibility study, the patient pathway was assessed through the patient retention rate at the end of the pilot.

The ability of the investigators to collect the data required for the case report forms was also assessed within the pilot trial; the end-points are both objective and subjective, requiring input from clinicians, patients and from trial care charts filled in by nursing staff. Throughout the trial, the successful completion and return rates of the case report forms will be measured and the return rate of all case report forms should be over 70%.

### Patient pathway

**Figure 1:**
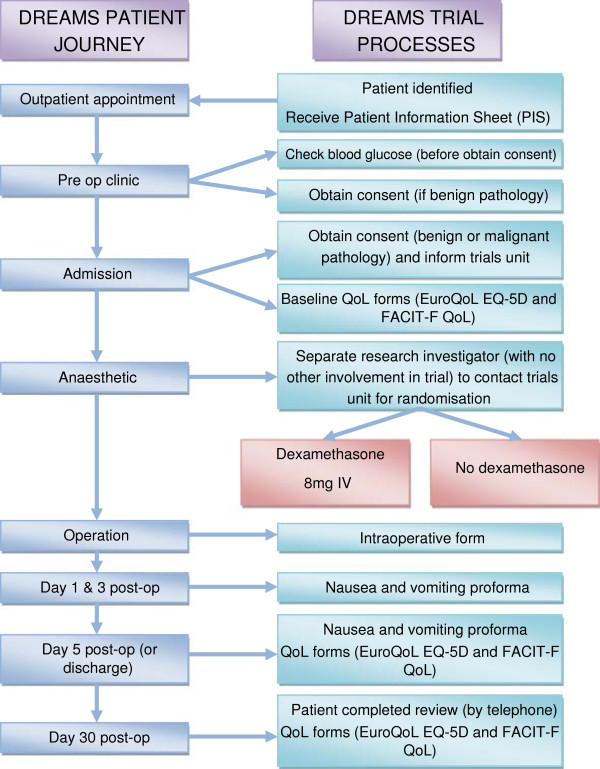
**Trial design and patient journey.** IV, intravenous; QoL, quality of life.

Patients will be seen by a member of the trial team (the Principle Investigator, Research Investigator or a Colorectal Nurse Specialist), who will discuss the trial with the patients and give them the ‘Patient Information Sheet’ and consent forms (Figure 1).

Patients will have the opportunity to further discuss the study at their preoperative assessment or on their day of admission with the Principal Investigator or Research Investigator at each hospital. The Principal Investigator or Research Investigator will obtain written informed consent.

On the day of admission and following consent for the trial, patients should be asked to complete the EuroQoL EQ-5D Quality of Life Questionnaire and the FACIT-F fatigue questionnaire.

All patients will be submitted to general anesthesia. This is not standardized in the trial. The randomized allocation will only be given to the anesthetist (or their operating department practitioner), following induction and after the administration of the one other antiemetic of the anesthetist’s choice. This is to maintain the double-blinding in the trial and to avoid bias in the anesthetic regimen.

Following surgery, patients will be given antiemetics as required. The choice of antiemetic will be as per local policy. Twenty-four hours post-operation, dexamethasone can be prescribed for the patient; however this must be on an ‘as required’ basis only. Dexamethasone must not be prescribed to a patient within the first 24 hours post-operation.

Follow-up data will include a nausea and vomiting review completed postoperatively at days 1, 3 and 5 (or day of discharge). This will include episodes of nausea and vomiting captured from patients’ care charts and antiemetic use captured from drug charts. Patients will also be requested to complete the validated Post Operative Nausea and Vomiting Intensity Scale at these time-points. Quality of life forms, including the FACIT-F Fatigue questionnaire, should be completed prior to surgery and then at 5 and 30 days postoperatively. An assessment of wound and chest infection as well as other complications during the postoperative period will be done via an outpatient appointment or telephone call 30 days postoperatively.

## Trial status

At the time of submission this trial is open to recruitment in 36 centers and has recruited 719 patients.

## Abbreviations

ERAS: Enhanced recovery after surgery; FACIT-F: Functional Assessment of Chronic Illness Therapy-Fatigue; IL: Interleukin; PCA: Patient-controlled analgesia; PONV: Postoperative nausea and vomiting.

## Competing interests

The authors declare that they have no competing interests.

## Authors’ contributions

RR is the Chief Investigator and has led in all stages of the study design. EHa, EHe, DB, MH, NM and SB all participated in the writing of the protocol, funding and ethics applications. AW provided anesthetic support for the study design. HBC is the trial manager and has assisted in site opening. KH provided the statistical support for the protocol. LM oversaw the ethics application, and assisted with writing the protocol and case report forms. DM has provided senior support throughout the design of the study and writing the protocol. All authors read and approved the final manuscript.

## Authors’ information

EH is Surgical Registrar at the West Midlands Deanery, Birmingham UK. RR is Registrar in General Surgery and Transplantation at the London Deanery, London, UK. DB is MRC Clinical Research Fellow at the NIHR Biomedical Research Unit and Centre for Liver Research at the University of Birmingham, Birmingham, UK. EH is a Clinical Research Fellow at the Centre for Liver Research, Infection and Immunity at the University of Birmingham, Birmingham, UK. MJH is Surgical Registrar at the West Midlands Deanery, Birmingham UK. NM is a Consultant Surgeon at New Cross Hospital, Wolverhampton, UK. SSB is a Vascular Research Fellow at St Georges Hospital, London, UK. AW is Consultant Anesthetist at University Hospital Birmingham, UK.HBC is Colorectal trial Co-ordinator at the Birmingham Clinical Trials Unit, University of Birmingham, UK. KH is Statistician at the Birmingham Clinical Trials Unit, University of Birmingham, UK. LM is Coloproctology Trials Team Leader at the Birmingham Clinical Trials Unit, University of Birmingham, UK. DM is Professor of Surgery at University Hospital Birmingham, UK.
